# Continuous prediction of glucose-level changes using an electronic nose in critically ill patients

**DOI:** 10.1186/cc13627

**Published:** 2014-03-17

**Authors:** JH Leopold, RT Van Hooijdonk, LD Bos, T Winters, PJ Sterk, A Abu-Hanna, MJ Schultz

**Affiliations:** 1 Academic Medical Center, Amsterdam, the Netherlands

## Introduction

Many if not most critically ill patients are treated with insulin during their stay in the ICU [1]. Intensive monitoring of the blood glucose level is a prerequisite for efficient and safe insulin titrations in these patients [2]. Current continuous glucose measurement techniques rely on subcutaneous glucose measurements [3] or measurements in blood [4]. We hypothesized that changes in volatile organic compound (VOC) concentrations in exhaled breath reflect changes in the blood glucose level. Changes in VOC concentrations can be analyzed continuously using a so-called electronic nose (eNose) [5]. Our aim was to investigate exhaled breath analysis to predict changes in glucose levels in intubated ICU patients.

## Methods

Exhaled breath was analyzed in 15 intubated ICU patients who were monitored with a subcutaneous CGM device. eNose results were compared with subcutaneous glucose measurements and linear regression models were built, including subject-specific models, and whole-sample models. The models were validated using temporal validation by training the model on the first 75% of measurements and prospectively testing on the last 25% of measurements. Performance of the models was measured using an R^2 ^value, Clarke error grids (CEG) and rate-error grid analysis (R-EGA).

## Results

Changes in VOC concentrations were associated with changes in subcutaneous glucose levels. R^2 ^performance had a mean value of 0.67 (0.34 to 0.98) for subject-specific models, and a mean value of 0.70.(0.52 to 0.96) for the model for the whole sample. However, when externally validating the model, the predictive performance dropped to a mean R^2 ^of 0.19 (0.00 to 0.70) for subject-specific models, and 0.04 for the model for the whole sample. Point accuracy in CEG was mostly good with >99% in zones A and B; trend accuracy, as visualized with R-EGA, was low.

## Conclusion

Exhaled breath prediction of glucose levels seems promising. However, performance of the current models is too low to be used in daily practice.
